# Focal Urethral Stricturing Following Intraurethral Mitomycin-C Gel and the Use of a Penile Clamp

**DOI:** 10.1155/2012/864741

**Published:** 2012-07-08

**Authors:** Richard F. J. Stanford, Stephen A. Thomas

**Affiliations:** Royal Derby Hospital, Derby Hospitals NHS Foundation Trust, Uttoxeter Road, Derby DE22 3NE, UK

## Abstract

We present a case of a 51-year-old gentleman, previously diagnosed with high-grade superficial transitional cell carcinoma of the bladder and treated with intravesical mitomycin C and BCG, who developed serial recurrences in the prostatic urethra. This was resected and treated further with intraurethral mitomycin-C gel. He subsequently developed an almost impassable distal penile urethral stricture, corresponding to the site of penile clamp application which we hypothesise is secondary to a combination of the mitomycin-C gel and penile clamp pressure.

## 1. Introduction

Transitional cell carcinoma (TCC) of the urethra is reported in between 12 and 40% of patients during surveillance following the diagnosis of TCC of the urinary bladder [1, 2], and in around 30% of patients undergoing radical cystoprostatectomy [3]; it is relatively rare for prostatic urethral TCC to be identified as an isolated, primary event [4]. Treatment usually consists of transurethral resection and can then be followed by treatment with intravesical agents such as mitomycin-C and Bacillus Calmette-Guèrin (BCG) (See review by Kirkali and Canda [5]). Whilst such intravesical treatments may confer benefit, there are reported risks to their use. We report a case where intraurethral mitomycin-C gel was used and retained in the urethra with the aid of a penile clamp and the patient subsequently developed a very dense, almost impassable distal penile urethra stricture corresponding to the site of clamp application.

## 2. Case Report

A 51-year-old type II diabetic gentleman had been under our care for one year with recurrent high-grade superficial transitional cell carcinoma (TCC) of the bladder. This had been treated with TURBTs and subsequent six instillation courses of intravesical mitomycin-C and BCG. He had, on three occasions, tumour in the prostatic urethra which was resected and then treated with three instillations, at three-week intervals, of mitomycin-C gel (20 mg Mitomycin-C in 5 mL water for injections and 5 mL Instillagel, that is, 2 mg·mL^−1^). Treatment was given for one hour, and the gel was retained with the assistance of a penile clamp. After the first treatment, the patient complained of severe dysuria, but after three weeks, continued with treatment. When readmitted for a check cystoscopy, he complained of continuing dysuria and deterioration in urinary flow. At cystoscopy he was found to have a very dense, almost impassable, distal urethral stricture which was eventually dilated using S-curve dilators over a guide wire up to 20 French to allow passage of the cystoscope. Cystoscopy demonstrated the stricture to be approximately 2.5 cm long and corresponded to the site and size of the penile clamp. There was no further stricturing and no evidence of urethral tumour recurrence proximally. Following a period of one month with an indwelling urethral catheter, his catheter was removed and he voided satisfactorily. He awaits further cystoscopic surveillance.

## 3. Discussion

TCC has been shown to be present in the prostatic urethra in around one-third of patients having cystoprostatectomy [3]. Patients with recurrent bladder TCC often receive intravesical chemo-/immuno-therapies which may not be in significant contact with the prostatic urethra. Previously, studies have shown that mitomycin-C gel instilled urethrally [6, 7] may be of benefit in reducing recurrence rates, although complications including pan-urethral structuring [8] have been reported. However, the type of stricture we have seen is different from those previously reported in that it is localised.

The role of mitomycin-C in the development of the stricture needs further consideration, and there is conflicting data with regard to mitomycin-C-related fibrosis. Short-term (five minutes) irrigation of the rat urethra following urethrotomy led to a reduction in histological findings with regards to fibrosis [9] and submucosal injection of mitomycin-C following urethrotomy has, in a small study [10], demonstrated a reduction in early restricturing rates. However, it is widely known that intravesical instillation of mitomycin-C can, rarely, cause severe perivesicular fibrotic reactions, and recently a study using an animal model has demonstrated that mitomycin-C can cause marked fibrosis within the bladder [11]. It is unclear why there is discrepancy between these studies; on one hand mitomycin-C is seen to reduce fibrosis, and on the other it is seen to increase fibrosis; such things as duration of exposure of the tissues to mitomycin-C and the concentration may need to be considered with regard to the effect on fibrosis.

We hypothesise that the cause of the focal structuring seen in this report is the combination of compression with the penile clamp and the presence of mitomycin-C. Our patient reported that he developed penile pain during, and for a time after, his first instillation of mitomycin-C gel. On subsequent occasions, he refused the use of the penile clamp and compressed his distal penile urethra manually.

In conclusion, we would suggest that careful consideration is given to the use of the combination of a penile clamp and mitomycin-C gel. A clamp giving a bare minimum of compression to retain the gel should be used so that no discomfort is felt; the gel should immediately be released if discomfort is felt.
